# Behcet Disease in pediatric Argentinian patients

**DOI:** 10.1186/1546-0096-13-S1-P170

**Published:** 2015-09-28

**Authors:** S Meiorin, C Vega, E Macias, T Gonzalez Vargas, G Espada

**Affiliations:** 1 Hospital de Niños Ricardo Gutierrez, Rheumatology, Ciudad Autónoma de Buenos Aires, Argentina

## 

Behcet's Disease (BD) is a multisystem inflammatory disorder of chronic course, highly frequent in certain ethnic groups (Turks, Israelis, Oriental). Although the disease is considered of low prevalence in children, recent registries indicates an increase in disease recognition. It characterizes by a broad spectrum of clinical manifestations, including ocular, musculoskeletal, CNS, gastrointestinal, vascular and mucocutaneous involvement. Its pathogenesis is very complex to be classified exclusively as an autoimmune or autoinflammatory entity. Objective: To describe the clinical and therapeutical characteristics of a series of patients with BD of onset in childhood. Patients and Methods: We reviewed the clinical charts of patients with BD age of onset ≤ 16 years, evaluated in our Rheumatology Section since 1994. The disease diagnosis was based on the International Study Group (ISG) criteria. Different variables were analyzed: demographic (including ancestry, age at presentation and delay time to diagnosis), clinical symptoms and affected organs, laboratory and therapeutic management (corticosteroids, immunosuppressive and biological agents) considering the response achieved. Results: 6 patients with BD were included, 4 females (66.7%) with mean age at onset of symptoms 9.05 years (range 4-12) and mean age at diagnosis 12.2 years (r 9-16). The average time of delay in diagnosis was 3.12 years. Turkish, Israeli and Armenian ancestry was found in 4 patients. No patient had positive family history of BD. Half of pts met criteria for classification of ISG. All patients had recurrent oral ulcers and 66.6% genital ulcers (n = 4) with no gender prevalence. Cutaneous manifestations were observed in 4 patients (66.6%) and consisted in erythema nodosum. The pathergy test was performed on 2 patients being negative. Four children presented uveitis (2 with hypopyon), and 2 of them received Infliximab as treatment due to a severe course (visual acuity 1/10). Clinical onset related to gastrointestinal symptoms (abdominal pain and enterorrhagia) was observed in 2 girls, associate in one case with neurological manifestations (pseudotumor cerebri). Molecular analysis was performed only in 1patient being HLA-B51 positive. All patients received steroids. Conclusion: In our series of patients with BD, the prevalence of clinical manifestations was similar to other series. Suspected symptoms of BD in the pediatric group are crucial for an early diagnosis and appropriate treatment.

**Table 1:** 

Pt	Age Dx / Gender	Oral/genital Ulcers	Compromised systems	Treatments	Response
1	16 / M	+/+	Eyes, skin, epididimitis	Methotrexate, Cyclosporine, Infliximab	Partial

2	12/F	+/+	Skin, CNS	Azathioprine, Mesalazine, Thalidomide	Complete

3	9/F	+ / +	Skin, eyes, Gastrointestinal	Azathioprine	Complete

4	10/F	+ / -	Eyes	Azathioprine, Infliximab	Partial

5	14/M	+ / +	Skin, eyes, arthritis	Colchicine, MTX, Azathioprine	Complete

6	12/F	+ / -	Eyes	Cyclosporine, Azathioprin	Complete

